# Short Delays in Time to First Contact With Community Health Services and Risk of Emergency Hospital Attendance: Retrospective Observational Study

**DOI:** 10.1111/jan.70488

**Published:** 2026-01-16

**Authors:** Lachlan Cameron, Matt Sutton, Beth Parkinson, Rachel Meacock

**Affiliations:** ^1^ University of Manchester Manchester UK

**Keywords:** avoidable hospitalisation, care delays, community health care, community nursing, emergency department

## Abstract

**Aim:**

To explore whether a delay from referral to first contact with nurse‐led community health services is associated with the likelihood of subsequent emergency department attendance.

**Design:**

We use individual linked administrative data on use of community health and hospital services. We identify a cohort of 343,721 individuals referred to community health services in England by their primary care provider in 2019. We then track their subsequent community healthcare contacts and emergency department attendances.

**Methods:**

We exploit variation in the time to contact caused by weekend delays, which create longer times to first contact for people referred later in the working week. The main analysis compares patients referred on Thursday with those referred on Tuesday.

**Results:**

We show that 6.7% of patients referred on Thursday wait an extra two days for their first community contact relative to those referred on Tuesday. Despite this delay, we find no evidence that people referred on Thursday are more likely to have a subsequent emergency department attendance compared to those referred on Tuesday.

**Conclusions:**

We do not find delayed community health services contact to be associated with an increased risk of emergency attendance amongst patients referred to community services by their primary care provider. This suggests that short delays in contact time are not detrimental for this group.

**Impact:**

Shifting care from hospital to community settings is a key priority for health systems internationally. In England, community health services face significant staffing shortages, limiting the extent to which services can be responsive and support the desired strategic shift. Our findings suggest that these constrained community providers could use their limited capacity to prioritise responding quickly to other patients without harming those referred via primary care.

**Reporting Method:**

STROBE guidelines.

**Patient or Public Contribution:**

This study did not include patient or public involvement in its design, conduct, or reporting.

## 1 Introduction

Worldwide, healthcare systems are in crisis with hospitals under increasing pressure due to large elective activity backlogs and unsustainably high bed occupancy (Samadbeik et al. 2024). In countries such as England, the plan is to reduce the burden on hospitals by shifting care to the community (NHS England 2025a). Despite policy ambitions that upscaling community health services (CHS) can reduce pressure on hospitals, there is currently very little evidence to support this (NICE 2018; Shi et al. 2024; Goff et al. 2025; Parkinson et al. 2025). One avenue through which community care is hypothesised to reduce the burden on hospitals is through intervening early to prevent deteriorations in health that would otherwise lead to avoidable emergency hospital presentations. This potential is highlighted in a recent study which estimated that 22% of emergency attendances are potentially avoidable (Jamieson et al. 2025). However, there are significant staffing shortages in CHS (Parkinson et al. 2024), which limits the extent to which these services can be responsive and support the desired strategic shift. Therefore, it is important to identify whether and for which types of patients CHS can avoid subsequent emergency hospital attendance, as this could identify opportunities to allocate the limited available community staff to the situations that would impact patients and hospital services the most.

## 2 Background

CHS for adults in England cover a wide range of services including district nursing, specialist long‐term condition nurses, occupational therapy, physiotherapy, and rehabilitation (NHS England 2025b). Whilst a range of staff are involved in the provision of CHS, 73% of clinical staff are nurses or nursing support staff (NHS England 2022), with the majority of services being nurse‐led. The sector plays a crucial role in treating and managing people's health needs in their homes and local communities, supporting people to live independently in their own homes and accounting for 13% of England's annual National Health Service (NHS) activity (The Kings Fund 2024). Services cover both step‐up care, supporting patients with care needs that have escalated beyond the level that can be managed in primary care, and step‐down care following hospital discharge (NHS England 2024).

A large body of literature has explored the impact of delays in receipt of step‐down care following hospital discharge, mainly examining outpatient and physician follow‐up, with mixed findings. Recent systematic reviews conclude that whilst the receipt of follow‐up care after discharge is generally found to be associated with a reduced risk of readmission, there is less evidence to support the importance of early timing of that follow‐up care for all patients (Bai et al. 2025; Balasubramanian et al. 2025). A meta‐analysis found evidence to support the importance of early post‐discharge follow‐up only for older patients and those with certain chronic conditions, recommending that early follow‐up care is therefore targeted at these high‐risk groups rather than universally implemented (Balasubramanian et al. 2025). Step‐down CHS care in England operates a targeted model, with acute providers referring patients who they deem to be in need to CHS following discharge. These patients then receive care from CHS in conjunction with outpatient and/or physician follow‐up, depending upon the reason for initial admission. There is, however, much less evidence on the impact of delays in care receipt earlier in the community care pathway, when patients are initially referred to CHS from primary care. It is unknown whether delays in care at this stage are associated with increased risk of hospital attendance.

## 3 The Study

This study aims to explore whether a delay in time from referral to first contact with CHS is associated with the likelihood of subsequent emergency department attendance. We focus on patients referred to CHS from primary care and explore whether those who experience a delay in having their first contact are more likely to have an emergency hospital attendance. This focus on primary care referrals allows us to explore the importance of timely CHS response in preventing initial emergency hospital presentation at the point when a patient's healthcare needs begin to escalate. We use individual‐level data on over 340,000 patients to consider how reduced availability of CHS at weekends leads to relative delays of one or two days in time to first CHS contact for people referred later in the working week and whether this subsequently predicts a higher likelihood of attending an emergency department in the 56 days after referral. CHS is a service with relatively quick response times, with 16% of our sample seen within one day of referral and nearly a third seen within one week, indicating that even a short delay of one or two days is substantial and could be detrimental for patients.

## 4 Methods

### 4.1 Data

We used patient‐level linked data for the financial year 2019/20 from two administrative sources: the Community Services Data Set (CSDS) and Hospital Episode Statistics (HES).

The CSDS is an individual‐level dataset to which all providers of NHS‐funded community health services in England are legally mandated to submit (NHS England 2025c). It captures information on referrals to, and contacts with, CHS providers, as well as patient demographic information including age, sex, ethnicity, and local area of residence (defined using Lower‐level Super Output Areas (LSOAs; ONS 2011)). For referrals, it contains information on the source of referral (e.g., primary care, hospital, self‐referral) and date of referral. For contacts, it contains information on the date of scheduled contact (if any) and if the patient attended the appointment (i.e., if care was delivered).

HES contains individual‐level information on the date and type of hospital presentations for patients at all NHS hospitals in England, with information submitted by the care provider (NHS England 2025d).

### 4.2 Sample

We examined a cohort of individuals referred to CHS by primary care providers between 1st May and 30th November 2019. This allowed us to adjust for healthcare use in the 28 days prior to referral and follow all individuals for 56 days after their referral, all within the financial year of data we use (2019/20). We excluded February and March 2020 as they may be impacted by the COVID‐19 pandemic.

Some CHS providers in England operate as stand‐alone community NHS Trusts, whilst others are combined with acute or mental health NHS Trusts (The Kings Fund 2024). We limited our sample to individuals referred to one of the 16 standalone CHS Trusts, as these large providers are more comparable in offering a full range of community health services.

The CSDS is a new data source and returns from some providers are known to suffer from omissions (NHS England 2025e). We excluded five providers with more than 10% missing data on the key variable that we use to identify our sample of interest: referral source.

We retained only first referrals within the period and excluded referrals of individuals with a hospitalisation on the same day as their referral (*n* = 6923). Our final analysis sample contained 343,721 individuals, of whom 69,461 were referred to CHS on a Tuesday and 68,880 were referred on a Thursday.

### 4.3 Variables

Our exposure of interest is the day of week of referral, and our primary outcome is emergency department attendance. We included only Type 1 emergency departments, which are 24/7 consultant‐led services available for all types of emergency patients with full resuscitation facilities. Our primary outcome is whether the individual had at least one attendance within 56 days of referral.

As control variables, we included sex and age (10 year bands) interactions, ethnicity (three major groups and a missing indicator), deprivation (quintiles of the Index of Multiple Deprivation (UK Government 2024)) and rurality of area of residence, disability status (indicator if the patient had been diagnosed with or self‐reported having a disability), priority type assigned by referrer (indicator if the service request was defined as clinically urgent by the referrer), four indicators for types of hospital contact in the 28 days prior to the referral (emergency department attendance, emergency admission, non‐emergency admission, and outpatient visit), and indicators for whether the individual received a referral to CHS from a source other than primary care or any CHS contact in the 28 days prior to our index referral date. We included age as a categorical variable to allow the relationship between hospital use and age to be flexible, capturing potential non‐linearities in this relationship.

### 4.4 Analysis

We exploited variation in the time to first CHS contact caused by weekend delays experienced by patients referred later in the working week. This avoids the potential for bias generated by unmeasured severity, which might lead to quicker first contacts and separately be associated with a higher risk of emergency department attendance.

#### 4.4.1 Establishing That Weekends Cause Delays

To demonstrate that patients referred later in the week have a longer time to first contact because of weekend delays, rather than patient severity, we first compared available characteristics related to patient severity by day of referral. We conducted pair‐wise tests for covariate balance, using linear regressions to test whether each of the covariates detailed in the Variables section above varied by day of referral. Because patient characteristics varied across providers, we included a set of provider indicators in these regression models to account for the CHS provider to which the individual had been referred (Kerwin et al. 2024). We then examined whether lower availability of CHS on weekends resulted in differences in the average number of days to first CHS contact between individuals referred on different days of the week.

#### 4.4.2 Analysing Whether Delays Increase Risk of Hospital Attendance

Subsequently, we estimated whether the likelihood of subsequent emergency department attendance differed for patients referred on days which are more impacted by weekend delays. For our main analysis, we restricted the sample to people referred on a Tuesday or a Thursday. Comparing these two days captures the full effect of weekend delays. It avoids including the few patients referred on weekend days, who have a higher measure of severity. It also avoids patients referred on Mondays and Fridays, who may be influenced by spillover effects from weekends.

For people referred on Thursday, the weekend creates a delay in time to first CHS contact for the patients who would have had their first contact two or three days after referral if they had instead been referred on a Tuesday. To focus on the patients most likely to be impacted by weekend delays, we stratified patients into four groups based on the number of days from referral to first contact: (i) contact on the same day or the day after the referral, (ii) contact within two to seven days of the referral, (iii) contact within eight to 28 days of the referral, and (iv) patients with no CHS contact within 28 days of referral.

We then estimated logistic regressions for emergency department attendance within each of the four groups, adjusting for the covariates detailed in the Variables section above. We also included a set of binary indicators for the CHS provider to which the patient was referred to account for unmeasured characteristics of providers that may impact both delays to care and the likelihood of a referred patient having an emergency department attendance.

We focused our main analysis on patients in Group (ii) (contacted within two to seven days of referral), because these are the patients experiencing the effects of the weekend delay. A higher likelihood of emergency department attendance for patients referred on Thursday relative to Tuesday amongst this group would be evidence that short delays in time to CHS care increase the likelihood of subsequent emergency department attendance.

The other three groups should not be impacted by initial weekend delays. Therefore, any difference between the likelihood of emergency department attendance between Tuesday and Thursday referrals in these groups might indicate significant differences in patient severity between referral days. We analysed these groups as a further check for significant differences in (unmeasured) patient severity between Tuesday and Thursday referrals.

Finally, we used a Mann–Whitney *U* test to check that day of referral does not impact selection into the four ‘time to contact groups’. This served two purposes: first, to provide additional evidence that patient severity was similar across referral days (i.e., higher likelihood of being in Group (i) for some referral days could indicate higher severity of patients); and second, to check for any anticipatory weekend effects if, for example, a higher proportion of patients referred on Thursday were seen within one day to avoid upcoming weekend delays.

All analyses were undertaken in R using the ‘fixest’ package (Berge et al. 2021), and we clustered the standard errors by CHS provider.

#### 4.4.3 Supplementary Analyses

First, we examined the sensitivity of our results to the definition of the outcome variable. Our primary outcome is whether the individual had at least one attendance within 56 days of referral. In supplementary analyses, we estimated models which defined the outcome as (i) emergency department attendance within 28 days of referral, and (ii) the count of attendances within 56 days. We used Poisson regression when analysing the count of attendances.

In other supplementary analyses, we compared all possible pairs of weekday referral days that are at least two days apart. We treated the referral day later in the week as the exposure. We also tailored our sample of interest to reflect when the referral day later in the week first experiences weekend delays, as we did in the main analysis by focusing on those whose first CHS contact was within two and seven days to reflect Thursday referrals first being impacted by weekend delays two days after referral. Here, in the analysis comparing Wednesday and Monday referrals, we focused on patients with their first CHS contact within three to seven days of referral since weekend delays first impact Wednesday referrals on day three. When comparing Thursday and Monday referrals, we focused on patients with their first CHS contact two to seven days after referral, as in the main analysis since Thursday is similarly the referral day later in the week. When comparing patients referred on Friday with patients respectively referred on Monday, Tuesday, and Wednesday, we focused on patients with their first CHS contact within one to seven days of referral, since weekend delays first impact Friday referrals one day after referral. In all these comparisons, a higher likelihood of emergency department attendance for people referred later in the working week would indicate that delays to CHS care increased the risk of emergency department use.

In a further supplementary analysis, we focused on three sub‐groups of patients where delays in care may be more likely to increase hospital use: (i) referrals classified as urgent by the referrer, (ii) referrals to a district nursing team (rather than teams such as physiotherapy or musculoskeletal, referrals to which are typically less time‐sensitive), and (iii) referrals for reasons classified by one of our General Practitioner (GP) colleagues as most likely to be sensitive to delays based on their clinical expertise and experience (listed in the Appendix Table A3). We conducted these analyses to explore whether delays to care impacted on specific sub‐groups of patients and these effects were diluted in the analysis of all patients.

In final supplementary analyses, we excluded patients referred in the weeks prior to and including the three Bank Holiday Mondays during our analysis period (6th May, 27th May, and 26th August). This was to check that our main results were not impacted by public holidays, when the availability of both primary care and CHS may be reduced.

### 4.5 Ethical Considerations

Ethics approval was not required because only non‐identifiable secondary use administrative data was used in this study. Completion of the Health Research Authority (HRA)'s online decision tool confirmed that NHS Research Ethics Committee (REC) review was not required. The research protocol did, however, undergo detailed review by NHS Digital before access to the Community Services Data Set (CSDS) and Hospital Episode Statistics was granted (DARS‐NIC‐482271‐S6S1V‐v0.4). Access to patient‐level CSDS and HES data is available only from NHS Digital through their Data Access Request Service (DARS) process, which required applicants to demonstrate that they meet strict data governance standards.

## 5 Findings

Table 1 presents descriptive statistics for each of the covariates by day of referral. The few people referred on a weekend were more likely to be older, from deprived areas, be assigned urgent priority by the referrer, and to have used health care services in the last 28 days compared to patients referred on weekdays. This indicates higher patient severity for patients referred on weekends. Patients were more similar between weekdays.

**TABLE 1:** Sample statistics by referral day of the week.

	Monday	Tuesday	Wednesday	Thursday	Friday	Saturday	Sunday
Number of times day of week occurred in our sample period	30	30	31	31	31	31	30
Observations	61,843	69,461	70,718	68,880	66,071	4072	2676
Mean age, years	64.6	64.9	64.8	64.8	64.7	69.1	71.0
Female	58.9	59.3	59.1	59.1	58.6	56.9	57.2
Ethnicity: White British	42.9	43.8	43.3	43.6	43.6	48.8	49.4
Ethnicity: White minorities	10.5	10.0	9.7	9.9	9.6	7.0	5.7
Ethnicity: other than White	5.5	5.4	5.3	5.1	5.6	7.0	6.3
Ethnicity: missing	41.1	40.8	41.7	41.4	41.2	37.2	38.5
Most deprived quintile	12.1	12.2	12.2	12.0	12.1	12.0	12.7
Second most deprived quintile	19.5	19.6	19.5	19.5	19.3	22.0	20.4
Middle deprivation quintile	26.0	25.6	25.7	26.0	25.9	26.5	25.4
Second least deprived quintile	21.7	22.2	21.9	22.0	22.1	21.6	22.8
Least deprived quintile	20.4	20.1	20.4	20.1	20.3	17.6	18.5
Rural	23.9	24.5	24.1	24.5	24.5	23.5	22.5
Any disability	6.0	6.0	6.1	6.2	6.2	6.9	5.5
Priority type assigned by referrer: urgent	9.6	9.3	9.2	9.5	9.5	16.9	20.3
*Healthcare interactions in the 28 days prior to referral, proportion having*							
Emergency department attendance	11.1	10.8	10.7	11.1	11.6	18.8	18.6
Emergency admission in 28 days prior	7.1	7.1	7.3	7.6	8.2	16.8	15.4
Non‐emergency admission	5.0	4.4	4.6	4.7	5.2	8.7	7.2
Outpatient attendance	26.3	26.0	26.5	26.5	27.0	30.0	27.4
Any CHS referral	8.0	8.5	9.2	8.9	8.9	16.2	18.3
Any CHS contact	12.0	12.7	13.4	12.9	13.1	23.6	27.1

*Note:* Values indicate proportion of sample unless specified otherwise (e.g., age presents the mean of a continuous variable).

### 5.1 Establishing That Weekends Cause Delays

Table 2 compares these statistics between Tuesday and Thursday, the referral days used in our main analysis. Column 4 reports the results of the pairwise covariate balance tests. Only one of the 20 covariates was statistically different at the 5% significance level: Thursday referrals had a 0.47 (95% CI: 0.20–0.73) percentage point higher likelihood of having had an emergency admission in the 28 days prior to referral. There is no consistent pattern across the covariate differences to indicate patients referred on one day are more severe on average than the other. Some characteristics suggest higher patient severity amongst Thursday compared to Tuesday referrals (e.g., slightly more likely to be assigned urgent priority by the referrer and to have a disability, and higher rates of prior hospital and CHS use in the 28 days before referral), while others indicate lower patient severity (e.g., younger, less likely to live in a deprived area). Overall, we find patients referred on Thursdays to be similar to patients referred on Tuesdays across a range of characteristics related to patient severity.

**TABLE 2:** Sample statistics, comparing Tuesday and Thursday referrals.

	Tuesday	Thursday	Unadjusted difference	Adjusted difference
Observations	69,461	68,880	—	—
Mean age, years	64.9	64.8	−0.03	−0.08 *[−0.32, 0.15] p = 0.44*
Female	59.3	59.1	−0.12	−0.12 *[−0.77, 0.53] p = 0.70*
Ethnicity: White British	43.8	43.6	−0.17	−0.20 *[−0.70, 0.29] p = 0.38*
Ethnicity: White minorities	10.0	9.9	−0.14	0.14 *[−0.23, 0.52] p = 0.42*
Ethnicity: other than White	5.4	5.1	−0.35	−0.21 *[−0.42, 0.00] p = 0.05*
Ethnicity: missing	40.8	41.4	0.66	0.27 *[−0.21, 0.75] p = 0.24*
Most deprived quintile	12.2	12.0	−0.17	−0.09 *[−0.48, 0.31] p = 0.63*
Second most deprived quintile	19.6	19.5	−0.09	0.04 *[−0.62, 0.70] p = 0.89*
Middle deprivation quintile	25.6	26.0	0.41	0.39 *[−0.10, 0.88] p = 0.11*
Second least deprived quintile	22.2	22.0	−0.23	−0.31 *[−0.71, 0.09] p = 0.12*
Least deprived quintile	20.1	20.1	0.01	−0.10 *[−0.53, 0.34] p = 0.63*
Rural	24.5	24.5	−0.03	−0.07 *[−0.33, 0.19] p = 0.56*
Any disability	6.0	6.2	0.15	0.08 *[−0.04, 0.20] p = 0.15*
Priority type assigned by referrer: urgent	9.3	9.5	0.21	0.07 *[−0.56, 0.70] p = 0.81*
*Healthcare interactions in the 28 days prior to referral, proportion having*				
Emergency department attendance	10.8	11.1	0.28	0.25 *[−0.11, 0.62] p = 0.15*
Emergency hospital admission	7.1	7.6	0.50	**0.47** ***[0.20, 0.73] p = 0.00***
Non‐emergency hospital admission	4.4	4.7	0.29	0.28 *[−0.06, 0.61] p = 0.10*
Outpatient attendance	26.0	26.5	0.49	0.45 *[−0.05, 0.94] p = 0.07*
Any CHS referral	8.5	8.9	0.39	0.38 *[−0.06, 0.82] p = 0.08*
Any CHS contact	12.7	12.9	0.15	0.17 *[−0.18, 0.51] p = 0.30*

*Note:* The unadjusted differences are presented in column (3). The adjusted differences in column (4) are coefficients on a Thursday indicator from linear regressions of the covariate that also include provider fixed effects. A positive value indicates a higher value for Thursday referrals compared to Tuesday. Standard errors are clustered by the CHS provider patients were referred to. 95% confidence intervals are presented in brackets. Bold indicates statistically significant difference at the 5% level.

The effects of weekends on CHS contact rates is clear (Table 3). There were similar rates of CHS contact on the same day as referral across weekdays. However, Friday referrals had less than half the rate of first contacts one day after referral compared with other weekdays (3.04% compared with 6.17%–6.40%), and Thursday and Friday both had lower rates (1.27% and 1.22%) of first contact two days after referral compared with the other weekdays (4.43%–4.85%). These patterns continued throughout the week as, for example, Tuesday referrals had a relatively low proportion of patients with their first CHS contact four and five days after referral, days which fall on the weekend.

**TABLE 3:** Number of days from referral to first CHS contact, proportions by day of referral.

Day of referral	Days from referral to first CHS contact									Not within 28
	0	1	2	3	4	5	6	7	8–28 combined	
Monday	9.84	6.40	4.73	3.29	2.19	0.21	0.11	1.74	14.38	57.11
Tuesday	10.07	6.29	4.85	3.23	0.29	0.14	1.97	2.15	14.15	56.86
Wednesday	9.58	6.17	4.43	0.48	0.27	2.30	2.65	2.33	13.78	58.03
Thursday	9.86	6.19	1.27	0.47	3.12	3.32	2.70	2.11	13.61	57.34
Friday	10.11	3.04	1.22	4.07	4.15	3.26	2.33	1.83	13.50	56.49

*Note:* Values indicate the proportion of referrals within each day of the week that receive their first CHS contact on the specified number of days after their referral (i.e., all rows sum to 1), where column 0 represents CHS contacts occurring on the same day as the referral. ‘8–28 combined’ denotes the proportion of referrals that receive their first CHS contact on any day between 8 and 28 days after referral. ‘Not within 28’ denotes the proportion of referrals that did not receive any CHS contact in the 28 days after their referral.

**FIGURE 1:**
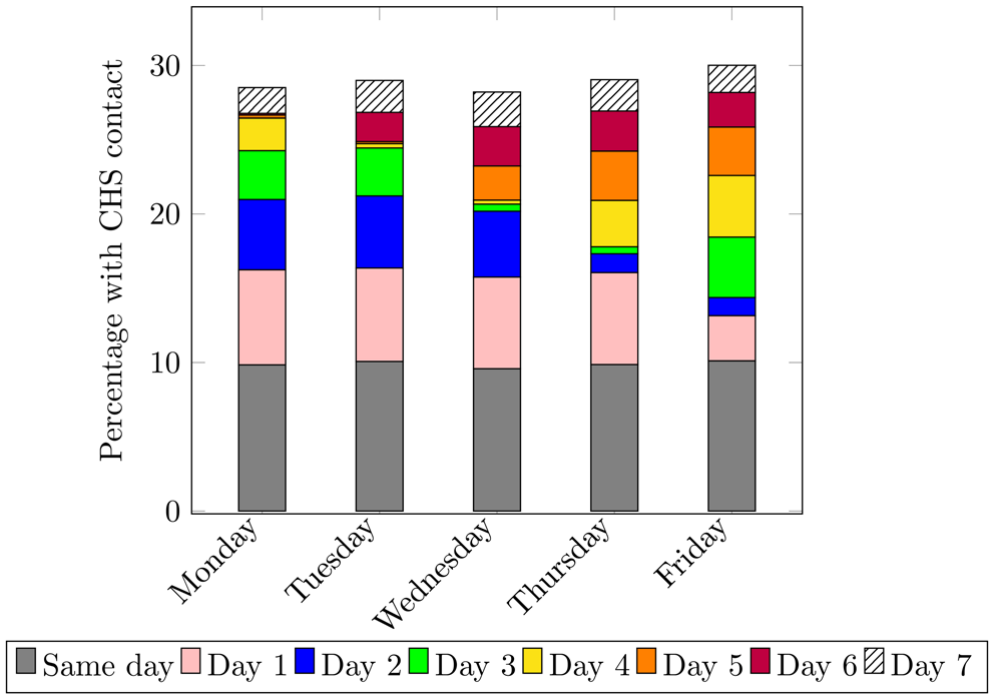
Distribution of time to first CHS contact by day of referral. The components of the bars represent the proportion of patients referred on the respective day of the week who have their first CHS contact on the specified number of days after referral.

Figure 1 presents these results graphically, showing the cumulative proportion of time to first contact by day of referral. Friday referrals had a lower likelihood of being seen within one day of referral relative to the other days, and both Friday and Thursday referrals had a lower likelihood of being seen within two days of referral relative to patients referred on Monday to Wednesday. Referral days earlier in the week had relatively few contacts on the days impacted by weekends. For example, Tuesday referrals have lower contact rates four and five days after referral. Rates of first contact returned to a similar level after days 6 and 7, once the follow‐up period for each referral day had included a weekend.

Table 4 presents the proportions of daily referrals in each of the four ‘time to contact’ groups for Tuesday and Thursday referrals. Using a Mann–Whitney *U* test, we found no statistically significant difference in the proportions of patients in these groups between Thursday and Tuesday. This also suggests patient severity is similar across Tuesday and Thursday referrals and indicates that there is no evidence of contact times being foreshortened in anticipation of the weekend.

**TABLE 4:** Difference in ‘time to contact’ groups between Tuesday and Thursday referrals.

	Proportion in each ‘time to contact’ group			
	0–1	2–7	8–28	Not within 28
Tuesday	16.36	12.63	14.15	56.86
Thursday	16.05	12.99	13.61	57.34
Difference	0.31	−0.36	0.54	−0.48

*Note:* Using a Mann–Whitney *U* test, we find no statistically significant difference in selection into these groups based on day of referral.

Table 5 reports the cumulative proportion of patients with their first CHS contact within a given number of days. The rate of CHS contact on the same day and the day after referral was similar for Tuesday and Thursday referrals. Thursday referrals had significantly lower rates of first CHS contact than Tuesday referrals on days two and three (1.74% compared with 8.08%), and much higher rates of contact on days four and five (6.44% versus 0.43%). The statistical significance between the days disappeared by day six and remained non‐significant for days seven and 28.

**TABLE 5:** Difference in cumulative distribution of time to first CHS contact between Tuesday and Thursday referrals.

Days from referral to first CHS contact										Not within 28
	0	1	2	3	4	5	6	7	28	
Tuesday	10.07	16.36	21.21	24.44	24.73	24.87	26.84	28.99	43.14	100
Thursday	9.86	16.05	17.32	17.79	20.91	24.23	26.93	29.04	42.66	100
Difference	0.21	0.31	**3.89**	**6.65**	**3.82**	**0.64**	−0.09	−0.05	0.48	—

*Note:* Bold indicates a statistically significant difference at the 5% level.

Together, Tables 4 and 5 show that the day of referral does not predict which of the four ‘time to contact’ groups patients are in, but that weekend delays cause differences in time to first contact between patients referred on Tuesday and Thursday specifically for the group with their first CHS contact within two to seven days of referral.

### 5.2 Analysing Whether Delays Increase Risk of Hospital Attendance

Column 1 of Table 6 presents estimates for the group of referrals for whom the weekend creates a delay in time to CHS contact for patients referred on Thursday relative to Tuesday. There was no significant difference (−0.0110, 95% CI: −0.0255 to 0.0036) between Thursday and Tuesday referrals in the adjusted probability of an emergency department attendance within 56 days amongst this group. This provides no evidence that short delays in time to CHS care increase the likelihood of subsequent emergency department attendance. The full model results, including estimated marginal effects of all control variables, and unadjusted results are presented in Appendix Table A5.

**TABLE 6:** Differences in emergency department attendance between Thursday and Tuesday referrals.

	‘Time to contact’ group hypothesised to be impacted by weekend delays	‘Time to contact’ groups *not* hypothesised to be impacted by weekend delays		
	First contact in 2 to 7 days	First contact in 0 to 1 days	First contact in 8 to 28 days	No contact within 28 days
Day of referral: Thursday	−0.0109	−0.0063	−0.0016	0.0028
	*[−0.0237, 0.0019] p = 0.10*	*[−0.0134, 0.0008] p = 0.08*	*[−0.0091, 0.0059] p = 0.68*	*[−0.0007, 0.0063] p = 0.12*
No. observations	17,722	22,422	19,205	78,992
Pseudo *R* ^2^	0.0368	0.0465	0.0674	0.0368
Control variables	YES	YES	YES	YES
Outcome mean	0.1715	0.2077	0.1139	0.0710
Regression model	Logit	Logit	Logit	Logit

*Note:* The models use logistic regression, and the average marginal effects are presented. Average marginal effects can be interpreted as the average percentage point difference in the likelihood an individual has an emergency department attendance (Norton et al. 2019). Covariates are: sex and age (10 year bands) interactions, ethnicity (three major groups and a missing indicator), deprivation (quintiles of the Index of Multiple Deprivation (UK Government 2024)) and rurality of area of residence, disability status (indicator if the patient had been diagnosed with or self‐reported having a disability), priority type assigned by referrer (indicator if the service request was defined as clinically urgent by the referrer), four indicators for types of hospital contact in the 28 days prior to the referral (emergency department attendance, emergency admission, non‐emergency admission, and outpatient visit), and indicators for whether the individual received a referral to CHS from a source other than primary care or any CHS contact in the 28 days prior to our index referral date. Models include a set of indicators for the CHS provider to which patients were referred, and standard errors were clustered by CHS provider.

There were also no differences in emergency department attendance rates between Thursday and Tuesday for any of the other ‘time to contact’ groups who are not impacted by weekend delays in CHS contact (last three columns of Table 6).

### 5.3 Supplementary Analyses

The results of our main model were robust to alternate specifications of the outcome variable (Table A1). There were no differences in the probability of emergency department attendance within 28 days (−0.0085; 95% CI: −0.0191 to 0.0022), nor the number of emergency department attendances in 56 days (−0.0435; 95% CI: −0.1271 to 0.0481).

In supplementary analysis comparing other pairs of referral days (Table A2), we again find no evidence of higher emergency department attendance amongst people referred later compared to earlier in the working week, despite those referred later experiencing a delay in time to first CHS contact. Estimates ranged from −0.0106 (95% CI: −0.0244 to 0.0033) when comparing Tuesday and Friday referrals to −0.0024 (95% CI: −0.0161 to 0.0113) when comparing Wednesday and Friday referrals, but none of the estimated effects were statistically significant.

We also find no differences in emergency department attendance rates between Thursday and Tuesday when restricting the sample to patients for whom we expect delays in care to have the most impact (Table A3). We find no evidence of differences between Tuesday and Thursday referrals for patients who are assigned urgent priority by the referrer (0.0190; 95% CI: −0.0059 to 0.0439), referred to district nursing (−0.0181; 95% CI: −0.0406 to 0.0043), or whose reason for referral was classified as potentially most sensitive to delays based upon the clinical experience of a GP (0.0031; 95% CI: −0.0227 to 0.0289).

Finally, our results are robust to the exclusion of referrals which might be impacted by bank holidays (Table A4). The estimates were similar to the main analysis, and none were statistically significant.

## 6 Discussion

### 6.1 Principal Findings

We explore whether short delays in time to first CHS contact predict a higher likelihood of subsequent emergency department attendance for people referred to CHS from primary care. We do this by exploiting variation in time to first CHS contact caused by weekend delays, whereby some people referred later in the working week experience a delay in receiving care because fewer care contacts are provided on weekends. We find no evidence that a short delay in time to first CHS contact predicts a higher likelihood of subsequent emergency department attendance.

Our main analysis compares emergency department attendance for people referred to CHS from primary care on Thursday with those referred on Tuesday. These populations have similar characteristics related to patient severity. The proportion of them that are first contacted by CHS on the same day and day after referral is also similar. However, fewer community staff working on weekends causes a delay in time to first CHS contact after the first day, such that Thursday referrals are less likely to be seen on day two or day three after referral. Despite this delay, we found no evidence of an impact on the risk of emergency department attendance. Extensive supplementary analyses confirmed the robustness of this finding that delayed first contact did not increase the risk of emergency department attendance. Null results remained for analyses with alternate measures of the outcome variable, comparing other pairs of referral days, restricting the sample to patients expected to be most impacted by delays, and excluding weeks before and after bank holidays.

### 6.2 Comparison With Previous Literature

The main contribution of this study is to improving understanding about whether CHS can reduce demand on hospitals, a topic with very little existing literature. A 2018 NICE review found some evidence that community nurse‐led care can reduce admission volumes, but studies were judged low quality (NICE 2018). Consistent with our null findings, area‐level analysis found no association between the provision of community nursing workforce and levels of hospital use amongst the older adult population they served (Parkinson et al. 2025). Given the methodology we employ, this study also contributes to literature on the effect of restricted weekend healthcare provision more generally. Most relevant to our study, Whittaker et al. (2016) find that extended access to primary care (at evenings and weekends) resulted in a 26.4% relative reduction in patient‐initiated emergency department visits for ‘minor’ problems. However, we find no evidence to suggest that extending weekend provision of CHS would impact emergency department visits.

Finally, this study contributes to the broader literature regarding delays to care. Evidence regarding step‐down care following hospital discharge has found that early follow‐up with outpatients can benefit high‐risk groups such as older patients and those with chronic conditions (Balasubramanian et al. 2025). However, consistent with our findings, evidence for the consequences of delays for the wider population is weak (Bai et al. 2025; Balasubramanian et al. 2025). Nevertheless, these reviews examined the impact of follow‐up care with outpatient or physician‐led services, as opposed to nurse‐led CHS care.

These and our findings contrast with literature from other settings, where detrimental consequences have been found as a result of delays in time to disease diagnosis (Hanna et al. 2020; Jayasooriya et al. 2023) and receipt of care in settings such as elective and emergency hospital care (Ciarleglio et al. 2021; Huang et al. 2010; Johnson et al. 2021; Oosterhoff et al. 2023). These differing conclusions may be due to differences in both the urgency of patients' clinical needs and the length of delays experienced across these different care settings.

### 6.3 Strengths and Limitations

We used a newly available large administrative dataset, which enabled linking of CHS referrals and contacts to hospital use for the first time in England. We examined over 340,000 individuals referred to multiple providers from different parts of the country, covering rural and urban areas. Furthermore, the administrative nature of the data means that the information is not self‐reported and hence not subject to recall bias or non‐disclosure problems. Since healthcare coverage is universal in England, insurance status and ability to pay do not influence CHS use or emergency hospital attendances.

This study is subject to some limitations. First, some variables in the CSDS contain missing values. In our sample, this impacted patient ethnicity, which we used as a covariate. We accounted for this in our analyses using the missing indicator method, in order to retain the full sample of observations. We also confirmed that levels of missingness were not correlated with day of referral, and hence are unlikely to have biased our results. Second, the dataset does not capture further use of primary healthcare, beyond the initial referral to CHS. Third, it remains possible that unmeasured aspects of patient severity differ between referral days. We demonstrate that measured patient characteristics were similar across referral days, with the exception of the rate of emergency admissions in the 28 days prior to referral, which was higher for Thursday compared to Tuesday referrals. A higher rate of prior emergency admissions could indicate greater patient severity, which would bias the risk of emergency department attendance for Thursday referrals upwards. Our finding of a null relationship between Thursday referral and emergency department attendance would therefore be more convincing evidence that short delays are not harmful. That said, our analysis only considers emergency hospital presentations as the outcome. We do not consider health outcomes or patient experience, which are not collected in the administrative datasets.

### 6.4 Implications

A priority of the NHS is to upscale provision of healthcare in community settings, with the intention of reducing demand on hospitals. However, there exists very little evidence for whether community health services can reduce subsequent emergency hospital presentations. This study provides unique insight into this. We find no evidence that short delays in time to first CHS contact are associated with an increased risk of emergency department attendance for people referred to CHS from primary care.

Patients referred to CHS for step‐down care following discharge are on average at higher risk of hospitalisation than those referred from primary care, with the 30 day emergency readmission risk following hospital discharge estimated to be 14% in England (Nuffield Trust 2022). It is possible that these other groups of patients may therefore be more sensitive to the timeliness of CHS care than those referred from primary care. Our results suggest that when faced with limited capacity, CHS providers could prioritise responding quickly to patients referred from other routes such as hospital, without increasing the risk of emergency hospital attendance amongst those referred via primary care.

However, we have not explored the impact of longer delays, and so would only recommend this response on the margin with short delays and for patients not meeting the threshold for same day CHS contact.

## 7 Conclusion

This study provides valuable insight into the sensitivity of emergency hospital presentations to timely CHS contact. We find no evidence that a short delay in time to first CHS contact increases the risk of emergency hospital presentations for people referred to CHS from primary care. Given the staff shortages in community health, it is important to make the best use of available staff. Our results suggest that community providers could use their limited capacity to prioritise responding quickly to other patients without harming those referred via primary care.

## Funding

This study is funded by the NIHR HS&DR programme (NIHR134436, R.M. recipient). This research was also supported by the National Institute for Health and Care Research Applied Research Collaboration Greater Manchester (NIHR200174, M.S. recipient). M.S. is an NIHR Senior Investigator. The funders had no role in study design, data collection and analysis, decision to publish, or preparation of the manuscript.

## Ethics Statement

Ethics approval was not required because only non‐identifiable secondary use administrative data was used in this study. Completion of the Health Research Authority (HRA)'s online decision toll confirmed that NHS Research Ethics Committee (REC) review was not required. The research protocol did, however, undergo detailed review by NHS Digital before access to the Community Services Data Set (CSDS) and Hospital Episode Statistics was granted (DARS‐NIC‐482271‐S6S1V‐v0.4).

## Conflicts of Interest

The authors declare no conflicts of interest.

## Data Availability

Data is not available without approval from the data holders.

## Appendix A

**TABLE A1:** Results with alternate measures of the outcome variable.

	Emergency department attendance in 28 days	No. emergency department attendances in 56 days
Day of referral: Thursday	−0.0082	−0.0431
	*[−0.0181, 0.0016] p = 0.10*	*[−0.1274, 0.0493] p = 0.35*
No. observations	17,722	17,722
Pseudo *R* ^2^	0.0353	0.0410
Control variables	YES	YES
Outcome mean	0.1087	0.2219
Regression model	Logit	Poisson

*Note:* The model in the first column uses logistic regression, with average marginal effects presented. The model in the second column uses Poisson regression, with the marginal effect presented (calculated by subtracting one from the exponential of the coefficient estimate, interpreted as the percentage difference in the individual's number of emergency department attendances). Models include fixed effects for the CHS provider patients were referred to, and standard errors were clustered by CHS provider.

**TABLE A2:** Different day comparisons, sample impacted by weekend delays (outcome is emergency department attendance within 56 days).

	Mon. v Wed.	Mon. v Thur.	Mon. v Fri.	Tue. v Fri.	Wed. v Fri.
Day of referral: Wednesday	−0.0093				
	*[−0.0231, 0.0045] p =0.18*				
Day of referral: Thursday		−0.0073			
		*[−0.0233, 0.0086] p = 0.37*			
Day of referral: Friday			−0.0036	−0.0106	−0.0024
			*[−0.0145, 0.0074] p = 0.52*	*[−0.0244, 0.0033] p = 0.14*	*[−0.0161, 0.0113] p = 0.73*
CHS contact sample	3–7 days	2–7 days	1–7 days	1–7 days	1–7 days
No. observations	10,331	16,533	24,687	26,290	26,310
Pseudo *R* ^2^	0.0428	0.0418	0.0358	0.0342	0.0350
Control variables	YES	YES	YES	YES	YES
Outcome mean	0.1624	0.1681	0.1846	0.1883	0.1856
Regression model	Logit	Logit	Logit	Logit	Logit

*Note:* Models include fixed effects for the CHS provider patients were referred to, and standard errors were clustered by CHS provider.

**TABLE A3:** Sub‐group analysis of patients hypothesised that a short delay in contact is most impactful for.

	Urgent priority	Referred to district nursing	Most sensitive to delays as judged by GP
Day of referral: Thursday	0.0190	−0.0181	0.0031
	*[−0.0059, 0.0439] p = 0.13*	*[−0.0406, 0.0043] p = 0.11*	*[−0.0227, 0.0289] p = 0.81*
No. observations	2261	7084	2208
Pseudo *R* ^2^	0.0478	0.0225	0.0110
Control variables	YES	YES	YES
Outcome mean	0.1685	0.1835	0.1970
Regression model	Logit	Logit	Logit

*Note:* These models are comparable to column 1 in Table 6: the sample is restricted to patients referred on Tuesday and Thursday and whose first CHS contact is within 2 to 7 days of their referral, and the outcome variable is a binary variable indicating if the patient has an Emergency Department attendance within 56 days of their referral. In addition, Column 1 only includes those whose referral is classified as urgent, Column 2 only includes those referred to a CHS district nursing team, and Column 3 only includes those whose primary reason for referral we deem important in terms of if a short delay is likely to impact them significantly (accident, bladder care, bowel problems, cancer, catheter problems, deep vein thrombosis, end of life support, equipment provisions, falls risk, family support, leg ulcer, mobility problems, pain control, post operative care, pressure ulcer, rehabilitation, and wound care). Models include fixed effects for the CHS provider patients were referred to, and standard errors were clustered by CHS provider.

**TABLE A4:** Results excluding weeks which might be impacted by bank holidays.

	Exclude week before bank holidays	Exclude week after bank holidays	Exclude both weeks
Day of referral: Thursday	−0.0103	−0.0123	−0.0117
	*[−0.0257, 0.0051] p = 0.19*	*[−0.0252, 0.0006] p = 0.06*	*[−0.0280, 0.0046] p = 0.16*
No. observations	16,122	15,513	13,913
Pseudo *R* ^2^	0.0351	0.0372	0.0350
Control variables	YES	YES	YES
Outcome mean	0.1709	0.1693	0.1684
Regression model	Logit	Logit	Logit

*Note:* Models include fixed effects for the CHS provider patients were referred to, and standard errors were clustered by CHS provider.

**TABLE A5:** Main model including coefficients on covariates.

	Unadjusted	Adjusted
Day of referral: Thursday	**−0.0124**	−0.0109
	***[−0.0242, −0.0005] p = 0.04***	*[−0.0237, 0.0019] p = 0.10*
Male, 45 and under		(Reference)
Male, 46–55		0.0097
		*[−0.0565, 0.0760] p = 0.77*
Male, 56–65		0.0486
		*[−0.0308, 0.1281] p = 0.23*
Male, 66–75		**0.0775**
		***[0.0052, 0.1499] p = 0.04***
Male, 76–85		**0.1461**
		***[0.0652, 0.2270] p = 0.00***
Male, 86 and over		**0.1130**
		***[0.0323, 0.1937] p = 0.01***
Female, 45 and under		0.0039
		*[−0.0405, 0.0484] p = 0.86*
Female, 46–55		−0.0102
		*[−0.0554, 0.0351] p = 0.66*
Female, 56–65		**0.0547**
		***[0.0065, 0.1030] p = 0.03***
Female, 66–75		0.0549
		*[−0.0053, 0.1151] p = 0.07*
Female, 76–85		**0.0893**
		***[0.0093, 0.1694] p = 0.03***
Female, 86 and over		**0.0904**
		***[0.0046, 0.1762] p = 0.04***
Ethnicity: White British		(Reference)
Ethnicity: White minorities		**0.0235**
		***[0.0015, 0.0456] p = 0.04***
Ethnicity: other than White		0.0142
		*[−0.0344, 0.0627] p = 0.57*
Ethnicity: missing		**−0.0264**
		***[−0.0432, −0.0096] p = 0.00***
Least deprived quintile		(Reference)
Most deprived quintile		0.0219
		*[−0.0048, 0.0486] p = 0.11*
Second most deprived quintile		0.0096
		*[−0.0055, 0.0247] p = 0.21*
Middle deprivation quintile		−0.0027
		*[−0.0168, 0.0114] p = 0.70*
Second least deprived quintile		−0.0071
		*[−0.0177, 0.0035] p = 0.19*
Rural		−0.0027
		*[−0.0158, 0.0103] p = 0.68*
Any disability		**0.0538**
		***[0.0266, 0.0809] p = 0.00***
Priority type assigned by referrer: urgent		−0.0062
		*[−0.0366, 0.0241] p = 0.69*
Emergency Department in 28 days prior		**0.0691**
		***[0.0504, 0.0878] p = 0.00***
Emergency hospital admission in 28 days prior		**0.0439**
		***[0.0162, 0.0715] p = 0.00***
Non‐emergency hospital admission in 28 days prior		−0.0053
		*[−0.0236, 0.0130] p = 0.57*
Outpatient in 28 days prior		**0.0421**
		***[0.0278, 0.0562] p = 0.00***
Any CHS referral in 28 days prior		**0.0354**
		***[0.0127, 0.0581] p = 0.00***
Any CHS contact in 28 days prior		**0.0198**
		***[0.0067, 0.0329] p = 0.00***
No. observations	17,722	17,722
Pseudo *R* ^2^	0.0034	0.0368
Outcome mean	0.1715	0.1715
Regression model	Logit	Logit

*Note:* Bold indicates statistical significance at the 5% level. Column 2 is the model in column 1 in Table 6: the sample is restricted to patients referred on Tuesday and Thursday and whose first CHS contact is within 2–7 days of their referral, and the outcome variable is a binary variable indicating if the patient has an Emergency Department attendance within 56 days of their referral. Column 1 presents the result of the unadjusted version of this model. Models include fixed effects for the CHS provider patients were referred to, and standard errors were clustered by CHS provider.
